# A Review of Unreduced Gametes and Neopolyploids in Alfalfa: How to Fill the Gap between Well-Established Meiotic Mutants and Next-Generation Genomic Resources

**DOI:** 10.3390/plants10050999

**Published:** 2021-05-17

**Authors:** Fabio Palumbo, Elisa Pasquali, Emidio Albertini, Gianni Barcaccia

**Affiliations:** 1 Department of Agronomy Food Natural Resources Animals Environment, Campus of Agripolis, University of Padova, 35020 Padova, Italy; fabio.palumbo@unipd.it (F.P.); elisa.pasquali.2@phd.unipd.it (E.P.); 2 Department of Agricultural, Food and Environmental Sciences, University of Perugia, 06121 Perugia, Italy; emidio.albertini@unipg.it

**Keywords:** *Medicago sativa*, meiotic mutants, 2*n* gametes, sexual polyploidization, diplospory, parthenogenesis, candidate genes

## Abstract

The gene flow mediated by unreduced gametes between diploid and tetraploid plants of the *Medicago* *sativa-coerulea-falcata* complex is pivotal for alfalfa breeding. Sexually tetraploidized hybrids could represent the best way to exploit progressive heterosis simultaneously derived from gene diversity, heterozygosity, and polyploidy. Moreover, unreduced gametes combined with parthenogenesis (i.e., apomixis) would enable the cloning of plants through seeds, providing a unique opportunity for the selection of superior genotypes with permanently fixed heterosis. This reproductive strategy has never been detected in the genus *Medicago*, but features of apomixis, such as restitutional apomeiosis and haploid parthenogenesis, have been reported. By means of an original case study, we demonstrated that sexually tetraploidized plants maintain apomeiosis, but this trait is developmentally independent from parthenogenesis. Alfalfa meiotic mutants producing unreduced egg cells revealed a null or very low capacity for parthenogenesis. The overall achievements reached so far are reviewed and discussed along with the efforts and strategies made for exploiting reproductive mutants that express apomictic elements in alfalfa breeding programs. Although several studies have investigated the cytological mechanisms responsible for 2*n* gamete formation and the inheritance of this trait, only a very small number of molecular markers and candidate genes putatively linked to unreduced gamete formation have been identified. Furthermore, this scenario has remained almost unchanged over the last two decades. Here, we propose a reverse genetics approach, by exploiting the genomic and transcriptomic resources available in alfalfa. Through a comparison with 9 proteins belonging to *Arabidopsis* *thaliana* known for their involvement in 2*n* gamete production, we identified 47 orthologous genes and evaluated their expression in several tissues, paving the way for novel candidate gene characterization studies. An overall view on strategies suitable to fill the gap between well-established meiotic mutants and next-generation genomic resources is presented and discussed.

## 1. Overview of the Occurrence of 2*n* Gametes and Their Use for Sexual Polyploidization in the *Medicago sativa-coerulea-falcata* Complex

One of the clearest and most immediate definitions of sexual polyploidization available in the literature was proposed by Mendiburu and Peloquin [1]. In this definition, sexual polyploidization is referred to as the process that leads to the formation of a euploid zygote from the natural fertilization events of restitutional 2*n* gametes (i.e., gametes with a somatic number of chromosomes). In contrast with zygotic and somatic chromosome doubling [2], the nonreduction of pollen grains and egg cells makes sexual polyploidization the prime mover of the origin and the evolution of polyploid plant species [3,4]. This important mechanism is the foundation of the cultivated alfalfa complex—the so-called *Medicago sativa-coerulea-falcata* complex that includes various outcrossing interfertile subspecies, either diploids (2*n* = 2*x* = 16) or tetraploids (2*n* = 4*x* = 32), sharing the same karyotype [5,6]. Some *M. sativa* spp. *falcata* and *M. sativa* spp. *coerulea* accessions are diploids, while some *M. sativa* spp. *falcata* and *M. sativa* spp. *sativa* accessions are tetraploids.

The coexistence of different levels of ploidy interfertile accessions, together with the occurrence of 2*n* gametes, generates new sexual polyploids, allowing for the flow of genetic resources and cultivar improvement in the cultivated alfalfa complex. Therefore, various authors have underlined the relevance of unreduced gametes in both the evolution [4,7] and the breeding [8,9,10,11,12,13] of alfalfa. In particular, the production of 2*n* gametes in *M. sativa* spp. *coerulea* and spp. *falcata* represents a powerful resource for the exploitation of continuous gene transfer from wild diploid forms to cultivated tetraploid alfalfa in assisted selection breeding programs. In fact, diploid meiotic mutant plants that are able to produce high frequencies of 2*n* pollen and 2*n* eggs have been widely exploited for unilateral directional introgression in interploid crosses in the *Medicago* complex [8,10,13,14,15].

**Figure 1:**
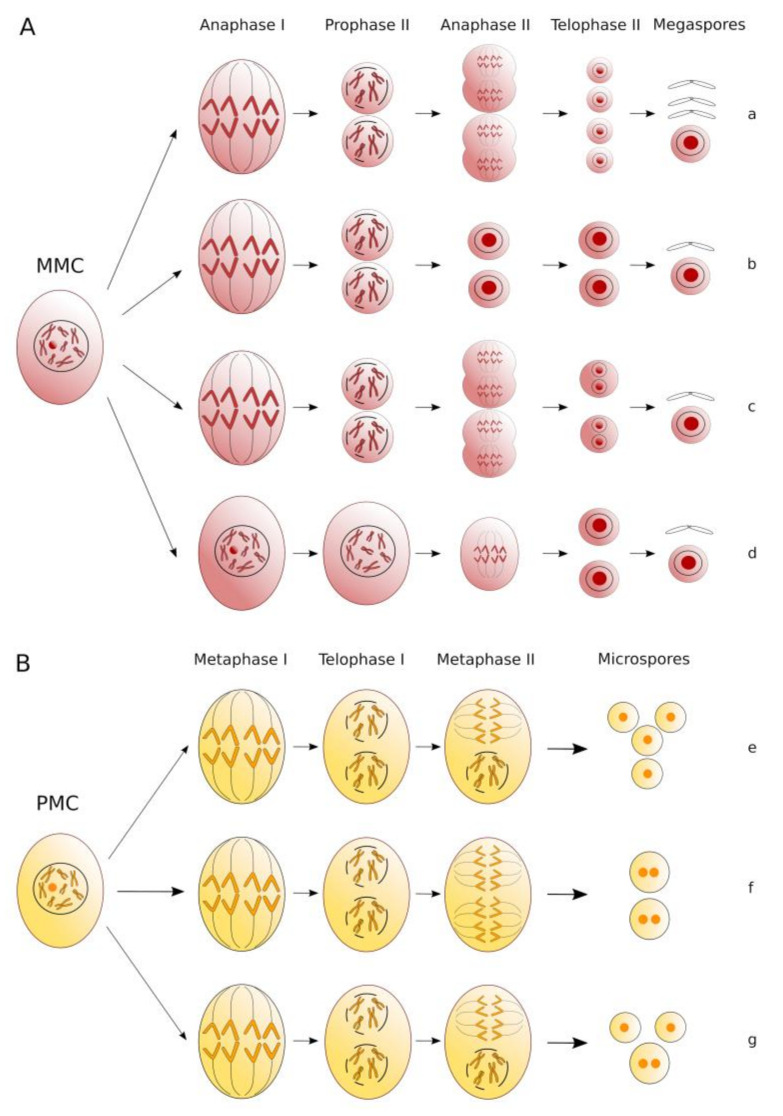
Schematic representation of the main defects of macro- (**A**) and microsporogenesis (**B**) processes that lead to 2*n* spore formation in alfalfa. Normal megasporogenesis producing a functional *n* megaspore (**a**); absence of the second meiotic division (**b**) and failed cytokinesis after telophase II (**c**) responsible for the 2*n* SDR megaspores; omission of reductional division followed by equational centromeric division that leads to FDR-type 2*n* megaspore production through diplosporic apomeiosis (**d**). Normal microsporogenesis resulting in a tetrad of *n* microspores (**e**); incorrect spindle orientation at metaphase II leading to two 2*n* microspores (**f**); null cytokinesis after telophase II responsible for the formation of a triad of one 2*n* and two *n* microspores (**g**) (this figure has been modified and adapted from [13]).

Among the various processes responsible for 2*n* gamete formation, several studies have proven that in alfalfa some cytological modifications that are genetically equivalent to first division restitution (FDR) and second division restitution (SDR) mechanisms lead to the production of unreduced pollen and eggs [16,17,18,19]. The main meiotic abnormalities responsible for the formation of 2*n* gametes are reported in Figure 1.

Since FDR-type 2*n* gametes are considered more advantageous than those obtained via SDR-type mechanisms [13,19], alfalfa breeding programs have focused on maximizing heterosis by transferring parental heterozygosity and retaining favorable epistatic interactions in the progeny via sexual polyploidization from the combination of FDR-type 2*n* eggs and FDR-type 2*n* pollen [8,20].

The cytological studies of reproductive *Medicago* mutants have allowed an in-depth investigation of the meiotic process. These studies culminated with the discovery that in this genus, meiosis is controlled by a great number of genes that are mostly present in a dominant state. [21]. Mutations in genes involved in meiosis cause deviation of the normal sporogenesis process, differentially affecting gamete formation and plant fertility and, therefore, creating new genetic variability. The possibility that the genes controlling meiosis are homologous between different organisms is highly likely, because the meiotic process is one of the most conserved biological phenomena in eukaryotes. Consequently, from an evolutionary perspective, mutations in these genes can also be considered homologous, having induced similar abnormal mechanisms in meiosis in different species.

The elucidation of the different steps of sporogenesis and gametogenesis has been possible due to the development of ovule- and anther-specific cytoembryological analyses based on stain-clearing and sectioning methodologies [15,22,23]. Although at low frequency, Clement and Stanford [22] reported the first observation of *2n* gamete occurrence on a 2*x* haploid individual of cultivated alfalfa. In particular, an anomalous cytokinetic process after telophase II of microsporogenesis was detected on the basis of unreduced pollen formation. Subsequently, fertile 4*x* tetraploid hybrid plants obtained from interploid matings of 2*x M. sativa* spp. *falcata*–4*x M. sativa* spp. *sativa* and reciprocals were used for further investigations of 2*n* gametes.

Studies performed on the progeny of these interploid crosses proved that 2*n* pollen that is genetically equivalent to that obtained after FDR mechanisms can be the result of an incorrect spindle orientation at metaphase II during microsporogenesis. Notably, spindles were found to be parallel to each other after abnormal cytokinesis, leading to the formation of dyads and sometimes triads. Moreover, it has been demonstrated that the creation of 2*n* microspores can also occur as a consequence of null cytokinesis after telophase II. In this case, the resulting dyads can be considered SDR-type 2*n* gametes [23]. Additionally, 4*n* or jumbo pollen formation has been shown to be due to the total lack of post-meiotic cytokinesis in alfalfa [24].

Early investigations on cytological mechanisms in the *Medicago* genus proved that the developmental stages in 2*n* egg formation are the same as those of *n* haploid megaspores during anaphase II. In the case of unreduced eggs, cytokinesis takes place only in the micropylar dyads and not in the chalazal dyads. Therefore, functional SDR-type 2*n* eggs remain at the chalazal end after micropylar megaspore disintegration [25]. Four different mechanisms are considered responsible for 2*n* egg formation: (1) failed cytokinesis after telophase II [23]; (2) the absence of the second meiotic division [17]; (3) the lack of the first meiotic division [26]; and (4) the irregularity that leads to FDR-type apomeiotic 2*n* megaspore production, as typically occurs in apomictic species [19,27].

As previously mentioned, the cytological aspects behind *2n* gamete formation have mainly been studied by exploiting meiotic mutants in 2*x*–4*x* and 4*x*–2*x* crosses in order to obtain tetraploid hybrids from interploid combinations. In the *Medicago sativa*–*coerulea*-*falcata* complex, a powerful triploid block gives rise to largely tetraploid progeny in interploid unions due to the abortion of almost all triploid embryos. Triploid elimination could be induced by abnormal endosperm development caused by the unbalanced 2:1 ratio between the maternal and paternal genomes [28]. Therefore, the number of seeds produced per pollinated flower, known as the seed set, in 2*x*–4*x* and 4*x*–2*x* crosses provides an assessment of the 2*n* gamete frequency generated by the diploid parents. Based on this index, it may be easier to select unreduced gamete-generating plants within diploid natural populations of *M. sativa* spp. *coerulea* and spp. *falcata* [10].

Additional parameters can be useful to promptly distinguish restitutional 2*n* gametes from reduced *n* gametes. In contrast with similar studies conducted in potato and red clover, in alfalfa it was found that the pollen grains of wild-type individuals are elliptical, while the pollen grains of meiotic mutants are mostly globular. Thus, it can be assumed that globular pollen is diploid and elliptical pollen is haploid [29]. In addition, since the nucleolar diameter of diplosporic cells was found to be on average 1.6-fold greater than that measured in normal *n* megaspores, the nucleolar dimension can be considered a reliable discriminating trait [15,17,19]. Nevertheless, cytological analysis combining nucleolar dimensions, integument growth, and cell appearance is still essential for an accurate evaluation of restitutional unreduced gametes in alfalfa.

The relevance of polyploidy is still a matter of debate within the scientific community. Some authors consider polyploid formation a simple and useless consequence of a “rare mitotic or meiotic catastrophe” [30] that traditionally leads to evolutionary “dead ends” [31], but thanks to recent genomic studies, polyploidy earned a key role in hybridization and speciation [32,33,34]. Several elegant studies have shown that polyploids confer three main advantages—namely, heterosis, gene redundancy, and asexual reproduction. If the first two are the effects of gene duplication, the way in which polyploidy affects sexuality is still unclear. In this way, polyploid organisms are more vigorous than diploids, creating novel genetic variation and covering the effects of deleterious recessive alleles and mutations [34,35]. In fact, the incidence of homozygous recessivity in polyploids is reduced by gene redundancy [36,37]; for instance, diploid *Aa* simple heterozygotes produce 1/4 *aa* homozygotes, autotetraploid *AAaa* biallelic duplex heterozygotes generate between 1/36 and 1/22 *aaaa* monoallelic quadruplex homozygotes, and allopolyploid *AaAa* double heterozygotes produce 1/16 *aaaa* double homozygotes [38].

Only a few studies have been undertaken to better understand the effect of polyploidization in autopolyploid species compared to allopolyploid species [39,40,41]. In addition, published results are mainly focused on polyploids obtained via somatic doubling, while the majority of polyploids in nature are produced by sexual mechanisms due to *2n* gamete production.

A recent study [42] shed light on the consequences of polyploidization on phenotype and gene transcription in alfalfa. In particular, the authors crossed *M. sativa subsp. falcata* seed parents and *M. sativa coerulea × falcata* pollen parents, two diploid meiotic mutants that are able to produce a mixture of reduced and unreduced gametes. Analyses were performed on full-sib 2*x* and 4*x* hybrids as the result of bilateral sexual polyploidization (BSP). In this way, the comparison between 2*x* and 4*x* progenies allows us to distinguish between the effects of intraspecific hybridization and those from sexual polyploidization. The superiority of 4*x* tetraploids to 2*x* diploid parents was found in several traits. Better leaf traits such as leaf size, epidermal cell surface, and stomatal size, along with higher green and dry biomass, resulted in better performances of tetraploid hybrids as a consequence of sexual polyploidization. Evidence of the increases in seed size (58%), autumn biomass (106%), canopy cover (30%), and leaf area (127%) under field conditions was more recently documented by Innes et al. [42]. Conversely, somatic doubling in alfalfa is not as related to polyploid superiority as was shown in maize, tobacco, and potato autopolyploids [39,41,43,44], confirming the maximization of heterozygosity as an effect of sexual polyploidization [45]. As expected from earlier studies [10,13], *4x* BSP hybrids presented a higher seed set than 2*x* hybrids. Thus, it is likely that sexual polyploidization positively affects fitness and reproductive performance in alfalfa, reducing disadvantageous parental alleles related to sterility [46]. Nevertheless, while examining anthers of some tetraploid *M. truncatula* plants, the authors reported events of pollen infertility caused by meiotic instability at the end of metaphase II [42]. Another adaptive advantage is represented by a higher seed set, which allows for the softening of summer drought effects that would be extremely damaging in the southern part of the distribution area of cultivated alfalfa [47]. Flowering time is another key trait in species adaptation. In the previously mentioned research [48], tetraploid progenies showed a shorter growth cycle, flowering earlier than diploid hybrids. Early flowering constitutes a primary trait in both natural conditions and cultivated fields. In the wild, it plays a fundamental role in competition with other species, while in fields, it has the double advantage of leading to higher biomass production and the ability to better outcompete weeds. Moreover, a relationship between seed size and early plant vigor in annual *Medicago* species was documented [42], underlining the possibility of improving seedling emergence in accordance with what was found for other species [49].

It is known from transcriptomic analyses that it is highly likely that sexual polyploidization influences gene expression, particularly that of genes linked to biotic and abiotic stress responses, energy metabolism, and plastid compartments. Further analyses found that some genes that encode lipoxygenases were overexpressed in *4x* BPS hybrids. Lipoxygenases (LOX) are responsible for the oxidation of polyunsaturated fatty acids to hydroperoxides and 13-LOX, particularly those widely involved in the jasmonate pathway. Due to the key role of jasmonate in plant stress responses, overexpression of LOX stands at the basis of a better adaptability of BSP autotetraploids in alfalfa. Similarly, two heat shock proteins (HSPs) were also overexpressed because of ploidy level changes, providing a more efficient response to heat and other abiotic stresses, as partially demonstrated in *Arabidopsis thaliana* allopolyploids [50]. Finally, better performances in terms of green biomass production can be explained by the upregulation of some photosynthesis-related genes. Rosellini et al. [48], in particular, found that the expression of genes encoding chlorophyll-binding proteins was crucial and directly involved in the capture and delivery of light excitation energy and the overexpression of the photosystem I subunit PsaD and the Rubisco small subunit.

Although *Medicago* species are widely recognized as the most relevant forage crops in the world and as model plants for other polyploid polysomic species, an information gap is still present. Sexual polyploidization along with *2n* gamete formation should not be overlooked as useful mechanisms in breeding and natural populations, especially with the currently available genomic and bioinformatic tools. In this review, we would like to stress the significance of sexual polyploidization for alfalfa breeding by showing some applicative examples of the exploitation of diplosporic tetraploidized mutants, and by providing genomic input for further research. Additional studies are also needed in order to clarify the genomic and transcriptomic mechanisms at the basis of sexual polyploidization. Meiotic mutants able to produce a consistent number of unreduced gametes may represent the most efficient means of exploiting and maximizing the evolutionary advantages of sexual polyploidization in alfalfa breeding.

The aim of this review is to pave the way for an innovative starting point in this specific research area, and to take a step towards the advancement of basic knowledge useful for alfalfa genetics and breeding. Here, we deal with a comprehensive report of all of the research activity conducted so far on unreduced gametes and neopolyploids in alfalfa, and we also present an original case study aimed at verifying the possibility of expressing somatic parthenogenesis in sexually tetraploidized plants, due to their natural ability to form apomeiotic gametes. An alternative strategy based on the most recent genomic and transcriptomic resources available for this species is then proposed for generating novel outputs and exceeding the practical limitations met so far in alfalfa breeding. Our mid-term goal is to fill the gap between well-established meiotic mutants and next-generation genomic resources in the *M. sativa*-*coerulea*-*falcata* complex.

## 2. Elements of Apomixis in Alfalfa: Sexually Tetraploidized Plants Maintain Apomeiosis Developmentally Independent of Parthenogenesis

As reported above, the *Medicago sativa* L. complex includes diploid and tetraploid sexual species that reproduce mainly through outcrossing, even if selfing is also possible. In polysomic polyploids such as cultivated alfalfa, maximum heterosis may be expressed by a few elite individuals of the population, but not by the entire population [51].

The potential of cloning plants through seeds offered by apomixis would provide a unique opportunity in cultivated alfalfa breeding for the selection of superior cultivars with permanently fixed heterosis. This reproductive strategy as a whole has never been detected in the *Medicago sativa-coerulea-falcata* complex, but features of apomixis—such as restitutional apomeiosis [19] and haploid parthenogenesis [52]—have been documented. In particular, cytological and molecular data have independently provided evidence that diplosporic mechanisms of unreduced egg cell formation occur in a diploid spontaneous mutant of *M. sativa* subsp. *falcata* named TNE (two-*n*-eggs). In addition, a progeny test based on morphological traits and molecular markers has indicated that apomeiosis in this case is not tightly associated with parthenogenesis [53].

Gametophytic apomixis has been shown to be strongly correlated with the occurrence of hybridity and polyploidy. Although numerous nonreductional meiotic mutants were described in the diploid forms of sexual species, the expression of apomixis was restricted mostly to polyploid apomictic complexes [54]. Therefore, the introgression of the diplosporic apomeiosis mutation at the tetraploid level could provide a novel opportunity to eventually induce somatic parthenogenesis through wide crosses with unrelated diploid materials.

**Figure 2:**
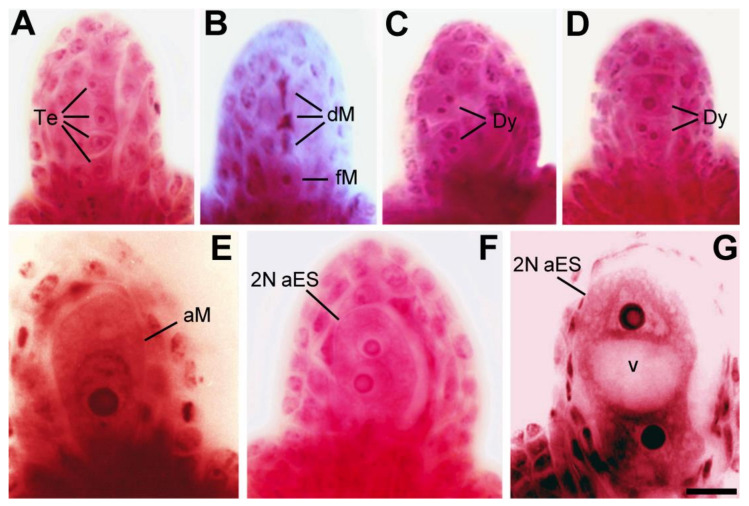
Alfalfa ovules analyzed by stain-clearing techniques, showing a linear tetrad of haploid megaspores (Te) derived from regular meiotic division (panel **A**), a chalazal regularly reduced functional megaspore (fM) along with three degenerating haploid megaspores (dM) (panel **B**), SDR-type dyads of megaspores (Dy) produced by the omission of cytokinesis after the second meiotic division (panels **C** and **D**), an enlarged uninucleate apomeiotic megaspore of the FDR-type (aM) derived from the lack of first meiotic division (panel **E**), and FDR-type unpolarized and polarized with central vacuole (v) binucleate apomeiotic embryo sacs (2N aES) (panels **F** and **G**) (these micrographs of ovules have been retrieved from [13,15,17,19] and graphically elaborated and standardized for magnification and micropylar (top) to chalazal (bottom) orientation by using Adobe Photoshop image analysis software; Bar = 10 μm in **A**–**D** and 5 μm in **E**–**G**).

At the University of Perugia and the University of Padova, bilateral and unilateral sexual polyploidization schemes have been adopted for introgressing diplosporic mutations at the tetraploid level. This was done by crossing the TNE mutant plants as females (2*n* egg cell producers) with 2*n* pollen producers of *M. coerulea* L. (2*n* = 2*x* = 16) as males (the experimental design related to this section is extensively described in Appendix A). The resulting tetraploidized F_1_ plants were then backcrossed as pollinators, with the TNE mutant producing unreduced egg cells in order to partly recover its genetic background and to assess the inheritance of the meiotic abnormality. This led to a yield of 33 seeds out of 2.619 pollinated flowers (Sed−Set = 0.0126). A total of 18 plants were recovered and screened for ploidy levels and occurrence of diplospory (Figure 2) by ascertaining their nuclear DNA content via flow cytometry analysis [55,56] Moreover, RAPD (random amplified polymorphic DNA) and AFLP (amplified fragment length polymorphism) markers were used for DNA fingerprinting [56,57], in order to discriminate plants of hybrid origin within the first generation back-cross or BC_1_ progeny, and to assess their genetic similarity with respect to the parental mutants.

Fifteen BC_1_ progeny plants were classified as tetraploid (Table 1) on the basis of their nuclear DNA content, which varied from 2.689 pg (plant B4) to 3.195 pg (plant B8) and was comparable to tetraploid values (plants coded CSE-1 = 2.610 pg and 10-TE = 2.996 pg), while three plants were classified as diploid, since their nuclear DNA contents were similar to that of TNE (1.446 pg), and were considered putatively to have arisen from selfing. In fact, these plants had most maternal RAPD and AFLP markers, and none of them was identical to TNE, proving that selfing and not parthenogenesis of 2*n* eggs was involved.

The diplosporic tendencies of BC_1_ plants were estimated by stain clearing as described in [56], and the degree of apomeiosis was calculated as the frequency of ovules with clear evidence of diplosporic cells or embryo sacs at stages from the megaspore mother cell (MMC) to the four-nucleated embryo sac (Figure 2). A total of 9 BC_1_ plants showed a reliable occurrence of diplosporic cells that ranged from 5.04% (plant C6) to 40.96% (plant B8), with an average of 17.72% (Table 1). The other six tetraploids showed a null degree of diplospory or a few and doubtful cases of apomeiosis (with a degree <2.44%, on average 0.76%). One of the three diploids (plant D3) showed a degree of diplospory as high as 57.89%. The nucleolar diameter of the diplosporic cells was on average 1.6-fold (i.e., 2.40 µm vs. 1.51 µm) that of the meiotic cells. Therefore, the authors considered the diameter of the nucleolus to be a good cell marker to discriminate diplosporic from meiotic cells (Table 1). Tetraploid plants with a diplosporic phenotype were then named diplosporic tetraploidized alfalfa (DTA).

Since a triploid embryo block that operates in interploidy crosses is effective in alfalfa, their ability to produce apomeiotic embryo sacs containing functionally unreduced egg cells at the tetraploid level was demonstrated by controlled crosses with octoploid pollinators of *M*. *sativa* subsp. *sativa*. The results demonstrated that the self-fertility of DTA plants was very low (0.007 on average), as was their cross-fertility with diploid testers (0.013 on average). However, considering the strong triploid block, which eliminates most triploid embryos, the seed set discrepancy of 0.006 observed between self- and cross-fertility with diploid pollinators could be attributable to parthenogenesis events primed by the wide crosses. The most promising DTA plant was B8, which showed (1) null self-fertility, (2) more than 40% diplospory, and (3) 1.8 seeds per 100 cross-pollinated flowers in 4*x*–2*x* crosses. The seed set values of DTA plants in controlled crosses with tetraploid testers (1.035 on average) indicated that regular meiosis occurred in these plants, and led to viable reduced egg cells (Table 1).

The aim of inducing the parthenogenetic development of unreduced egg cells was fulfilled by auxin treatments and by wide crosses using pollen of completely unrelated species, as reported in [56]. DTA plants produced seeds from both auxin treatments and wide crosses (Table S1). In particular, 4 plants yielded an average of 5.7 seeds out of 47 flowers treated with NAA (naphthalene acetic acid), whereas 2 plants produced an average of 5.6 seeds out of 46 flowers pollinated. These seeds were sown, and DNA from each plant was analyzed using PCR-based molecular markers. Molecular progeny tests indicated a sexual rather than apomictic origin of DTA progeny, even if several plants showed no molecular differences with respect to the mother plant [56]. Therefore, the fact that DTA plants set only a few seeds in either wide crosses or auxin tests is evidence of a null or very low capacity for parthenogenesis of their unreduced egg cells.

## 3. Candidate Genes for Unreduced Gamete Production and Exploitation of Genomic Resources for the Analysis of Alfalfa Meiotic Mutants

Diploid meiotic mutants producing viable and unreduced (*n* ≥ 2) gametes have been discovered in numerous plants [58], and although complete penetrance is extremely uncommon, it is thought to be an almost ubiquitous behavior across the entire plant kingdom [59]. Practically speaking, unreduced gametes are largely employed for polyploidization processes, and to boost the heterosis effect in several crops, including alfalfa [51,60]. Overall, male and female gamete production is controlled by a complex gene network, but usually *n* ≥ 2 eggs/pollen are the result of single gene mutations and, consequently, of a loss of protein function that may occur from early meiotic events (prophase I) to cytokinesis II [61]. In particular, when meiosis I fails to separate homologous chromosomes, we speak generally of “first division restitution” (FDR), while when meiosis II fails to separate sister chromatids, we refer to “second division restitution” (SDR) [59].

### 3.1. Forty Years of Genetic Resources and Genomic Studies in Medicago sativa

The exploitation of genetic and genomic resources epitomizes the main screening tool to reveal potential candidate genes controlling unreduced gamete formation. Before the advent of omics technologies, breeding schemes, linkage map construction, and marker-phenotype associations represented elite systems to narrow down the genomic regions involved in unreduced gamete production. This has been possible thanks to both the interfertility existing among the *Medicago sativa* subspecies (*sativa*, *coerulea,* and *falcata,* also known as the *Medicago* complex [62]), and the discovery of diploid genotypes producing 2*n* gametes (mainly isolated from *coerulea* and *falcata* [13]).

Since the 1980s, the constitution of ad hoc experimental populations by selfing and crossing (F_2_) or backcrossing (BC_1_) has helped to partially elucidate the segregation patterns of genes putatively involved in 2*n* pollen formation. Among the major findings, a single recessive gene (designated restitution pollen, *rp* [18]) was thought to be responsible for FDR 2*n* male gamete formation, while a second recessive gene (named jumbo pollen, *jp* [24]) involved in post-meiotic cytokinesis seemed to be the cause of 4*n* male gametes. However, the lack of molecular data and tools able to discriminate genotypes producing n gametes from those producing unreduced gametes made the exploitation of this information, both for breeding purposes and for basic research, extremely complicated.

A few years later, the advent of PCR increased the opportunities offered by molecular biology and, through the construction of molecular-marker-based linkage maps, allowed for a more gene-focused approach. With this aim, throughout the 1990s, several genetic linkage maps were developed in *Medicago sativa* by using, alone or in combination, molecular markers such as the RFLP, RAPD, AFLP, and SSR markers [63,64,65,66]. Among the most relevant findings, a Vg1G1b RFLP marker, located in linkage group 6, was found to be tightly associated with the *jp* (jumbo pollen) phenotype at a two-point distance of 2.4 cM [67], while a 610-bp ISSR marker amplified with a (CA)_8_GC primer was located 9.8 cM away from a locus controlling 2*n* egg formation (*tne*, *two-n egg* mutant gene [68]).

The earliest candidate genes in *M. sativa* that paved the way for functional genomic studies were identified at the beginning of the 2000s. One of the first genomic loci thought to be possibly responsible for 2*n* egg formation is a β-tubulin-codifying gene (GenBank ID AJ319667) [69]. Incorrect orientation of spindle formation and altered chromosome segregation, of which β-tubulin is one of the leading actors, have been directly correlated with unreduced gamete production (for more details, see [61,70]) in both meiosis I (e.g., wheat-rye hybrids [71]) and meiosis II (e.g., white poplar [72]). Among the clues supporting this hypothesis in alfalfa is an altered level of expression in 2*n* egg mutants compared to wild type [69,73], and a significant abundance of β-tubulin transcripts in the outer cell layer of ovules and in functional megaspores [74]. However, despite this histological and transcriptional evidence, a proper functional characterization of this gene proving its direct involvement in unbalanced gamete formation is still lacking in alfalfa.

A second candidate gene, a member of the *Mob1* (*Mps one binder*)-like family, received far more attention [69]. Before the discovery of *Mob1*-like genes in plants, studies performed in yeast [75,76] proved the involvement of some of these genes in cytokinesis and mitotic exit, while some *Drosophila* and human homologs (i.e., the dMob1 and *hMob1* families, respectively) seemed to regulate cell proliferation [77]. In a first study, multiple copies of *Mob1*-like genes were identified in the *M. sativa* genome through Southern blot, while through a Northern blot hybridization assay in mutant plants, Mob1 transcripts were exclusively located in flower buds at the early stages of meiosis, and not in any of the other vegetative organs [69]. Further mRNA localization and protein immunolocalization assays confirmed the presence of *Mob1*-like transcripts and gene products in degenerating megaspores of normal ovules, and in the enlarged megaspore mother cells and embryo sacs of apomeiotic ovules [78]. Finally, subcellular localization studies proved the involvement of these proteins in cell proliferation and, in particular, in cytokinesis. In fact, while their cytoplasmic localization was faint and diffused in the G1 and S phases, a progressive concentration of these proteins in fibrillar structures was observed during the G2 and M phases. At the cytokinesis stage, the protein was found to co-localize with microtubule structures at the emerging cell plate and near the septum [79].

In recent years, with the advent of omics approaches, the amount of molecular data available for *M. sativa* has increased exponentially in terms of both transcriptomics and genomics. A first attempt to analyze transcriptomic data in this species was performed in 2009, when the Medicago GeneChip array—originally developed for *Medicago truncatula*—was successfully tested on two alfalfa genotypes in order to identify the loci responsible for stem cell wall lignin and cellulose concentrations [80]. The first comprehensive alfalfa transcriptome was generated a few years later using 27 *M. sativa*, *coerulea*, and *falcata* genotypes: 870,000 SNPs were identified and proposed for marker-assisted breeding strategies [81]. In a second study, the 9000 most polymorphic SNPs (selected from [81]) were chosen to develop the first alfalfa SNP array, and were further validated in 280 alfalfa genotypes, again belonging to the *M. sativa*, *coerulea*, and *falcata* complex [82]. In both studies, a conspicuous number of SNPs was found to differentiate *M. falcata* (diploid and tetraploid), *M. coerulea* (diploid), and *M. sativa* (tetraploid). However, since the ploidy level of the gametes produced by each investigated genotype was unknown, it was not possible to associate any of these SNPs to *n* ≥ 2 gamete formation. Almost all of the transcriptomic studies subsequently conducted on alfalfa addressed resistance to biotic and abiotic stresses, or the specific pathways directly or indirectly involved in biomass production. Among them, we highlight some efforts to disclose the molecular mechanisms underlying freezing stress [83], waterlogging [84], salt [85], aluminum [86], nematode resistance [87], fall dormancy [88], and cell wall composition [89]. Finally, the gene expression atlas was produced from two alfalfa subspecies: *M. sativa* ssp. *sativa* (B47) and *M. sativa* ssp. *falcata* (F56). In contrast to previous studies, where leaves or stems were the only tissues considered, transcripts from six different tissues were sequenced, and their expression levels were compared [90].

From a genomic point of view, the publication of the first high-quality alfalfa genome sequence occurred only in 2020 [91]. An allele-aware chromosome-level genome assembly of a tetraploid genotype consisting of 32 allelic chromosomes and 138,729 homologous genes was produced by integrating high-fidelity single-molecule sequencing and Hi-C data.

Despite the genome assembly release and the remarkable number of gene expression atlases, studies aimed at identifying genes responsible for the formation of unreduced gametes have remained almost unchanged over the last 15 years. Moreover, although the number of studies supporting the preeminent role of epigenetics in gamete formation is rapidly increasing in other model species, in alfalfa there seems to be a total lack of literature on this subject.

Therefore, unlike the studies carried out thus far on alfalfa, based on a forward genetics approach (i.e., from phenotype to genotype), in the following section, we propose a reverse genetics approach (i.e., from genotype to phenotype) by exploiting the genomic and transcriptomic resources available, similar to what has been previously done in other species [92,93,94].

### 3.2. Identification of Candidate Genes through a Reverse Genetics Approach

To take the first step in this direction, we first identified nine well-characterized proteins belonging to *Arabidopsis thaliana*, known for their direct involvement in unreduced gamete production and never studied in alfalfa, from the scientific literature. Among the genes responsible for FDR, *SWITCH1*/*DYAD, ATPS1, JASON, ATSPO11-1,* and *REC8* were selected. *SWITCH1(SWI1)*/*DYAD* is one of the most pivotal genes for the proper course of prophase (I) and, in particular, for chromatid cohesion and recombination. In arabidopsis, loss of function of *SWI1/DYAD* is responsible for premature separation of sister chromatids [95] (normally occurring in anaphase (II), and mainly affects female meiosis. Although in some cases this leads to a blockage of female meiosis and to nonfunctional female gametophyte production [96], in others, unreduced (2*n*) female gametes were observed and exploited with reduced (*n*) male gametes for triploid (3*n*) seed production [97]. *ATSPO11-1* seems to be involved in crossover events, as failure to make double-stranded breaks and the absence of recombination are observed in arabidopsis *atspo11-1* mutants. Consequently, univalents segregate randomly during meiosis I, and unbalanced meiotic products are formed after meiosis II [98]. *REC8 (MEIOTIC RECOMBINATION PROTEIN REC8)* is necessary both to maintain centromere cohesion at anaphase I and for the monopolar orientation of the kinetochores during the first meiotic division [99]. *atps1* (*parallel spindle 1*) mutants are instead characterized by abnormal spindle orientation at male meiosis II, leading to diploid pollen grain formation and, thus, spontaneous triploid plants in the following generation [100]. Similar to *ATPS1*, the *jason* mutant in Arabidopsis is responsible for the production of unreduced first division restitution spores, because of the formation of parallel arranged and fused spindles during male meiosis II [101,102]. Moreover, although the role of this protein in male meiosis is not fully clear, by means of transcriptomic data, it has been hypothesized that the JASON protein is able to regulate *ATPS1* expression.

Regarding the genes involved in second division restitution, *CYCA1;2/TAM, CDKA;1*, *GIG1/OSD1*, and *NACK2* were chosen. In *cyca1;2/tam* (*cyclin-a 1;2/tardy asynchronous meiosis*) mutants, meiosis II is skipped, and sister chromatids remain attached. The resulting unreduced gametes are viable and contain both sister chromatids [103,104]. This protein, a member of the cyclin A family, is also thought to modulate the activity of (i.e., it presumably activates) a second gene, *CDKA;1 (A-TYPE CYCLIN-DEPENDENT KINASE),* whose product represents a key kinase involved in meiotic progression. Although *cdka;1* knockout mutants are lethal to the embryo, a weakly altered functionality (characterizing the T161D CDK variant) produces meiotic defects and, in some cases, unreduced gametes [105]. Another gene playing a crucial role in meiotic cell cycle progression is *OSD1* (*OMISSION OF SECOND DIVISION 1*), also known as *GIG1* (*GIGAS CELL 1*), a *UVI4*-like gene *(UV-B-INSENSITIVE 4-LIKE*) that is extremely conserved throughout the plant kingdom and has never been identified in other kingdoms. The *osd1* mutants experience normal chromosome segregation in meiosis I, but fail to enter the second meiotic division, leading to 2*n* eggs and *2n* pollen production [106]. Finally, *NACK2* (*NPK1-ACTIVATING KINESIN 2*, better known as *TETRASPORE*/*STUD, TES*/*STD)* is a predicted kinesin that positively regulates cell plate expansion. In Arabidopsis mutants, microtubule disorganization is observed after the second meiotic division, hindering the cytokinesis process. Four nuclei were therefore confined to the same cytoplasm, and some of them fused before the occurrence of the first mitotic division, leading to pollen grains with diploid, triploid, or even tetraploid nuclei. In contrast, reduced egg production proceeds regularly [107].

Forty-seven amino acid sequences from a recently assembled autotetraploid (2*n* = 4*x* = 32) alfalfa proteome [91] were selected based on their putative orthology (BLASTp; http://blast.ncbi.nlm.nih.gov/Blast.cgi (accessed on 15 March 2021), E-value from 5 × 10^−30^ to 0) with the nine Arabidopsis proteins described above (Table 2 and Table S2).

**Figure 3:**
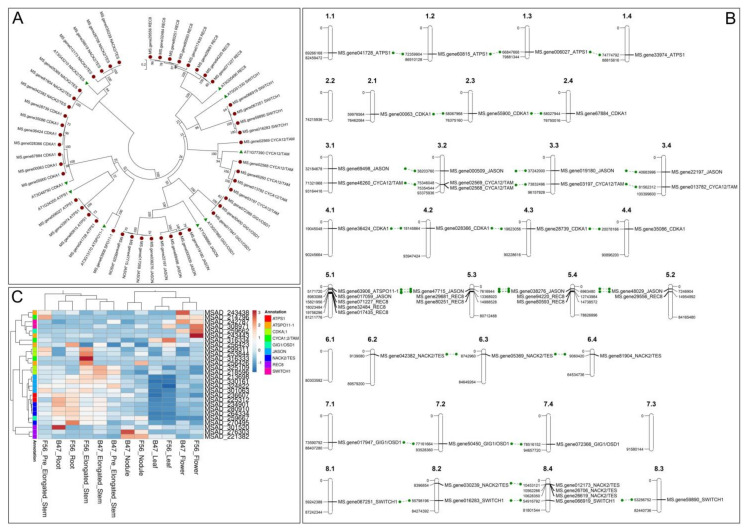
Similarity-based UPGMA analysis performed using 47 amino acid sequences from the *M. sativa* proteome [91] and selected for their putative orthology (Table 2 and, more specifically, Table S2) with 9 well-characterized proteins belonging to *A. thaliana* (At) selected for their direct involvement in unreduced male and/or female gamete production (**A**). Distribution of the 47 candidate genes throughout the 8 chromosomes of a tetraploid alfalfa genotype. Green lines connect allele copies of homologous chromosomes (**B**). Taking advantage of a recent gene atlas available for alfalfa [90], the relative abundance levels of 28 transcripts putatively corresponding to the 47 protein sequences mentioned above (BLASTp; http://blast.ncbi.nlm.nih.gov/Blast.cgi, (accessed on 15 March 2021; Table 2 and Table S2) were evaluated in 6 different tissues (roots, root nodules, leaves, flowers, elongating stem internodes, and post-elongation stem internodes) and two genotypes (B47, *M. sativa* ssp. *sativa* and F56, *M. sativa* ssp. *falcata*) (**C**). Expression data are available in Table S3.

A ClustalW alignment followed by a similarity-based UPGMA (unweighted pair group method with arithmetic mean) analysis (MEGA 7.0.26) was then performed using the amino acid sequences of both species (*Arabidopsis* and alfalfa, Figure 3A). The phylogenetic tree demonstrates that the sequences selected from Arabidopsis clustered together with the putative alfalfa protein orthologs, with bootstrap support values ranging from 72 to 100. According to their chromosome location, the 47 alfalfa sequences were also mapped and graphically represented throughout the 8 linkage groups (Figure 3B) to highlight the allele copies available for each of the four homologous chromosomes. Overall, for each Arabidopsis gene, we identified a number of putative alfalfa orthologs ranging from 1 (*ATSPO11-1*) to 8 (*JASON* and *REC8*). Except for *ATSPO11-1* and *GIG1/OSD1*, the four allelic forms (one for each homologous chromosome) were retrieved for each gene.

Taking advantage of the most comprehensive gene atlas available for alfalfa [90], the relative abundance levels of 28 transcripts putatively corresponding to the 47 protein sequences mentioned above (BLASTp; http://blast.ncbi.nlm.nih.gov/Blast.cgi, access on: 15 March 2021; Table 2 and Table S2) were evaluated in 6 different tissues (roots, root nodules, leaves, flowers, elongating stem internodes, and post-elongation stem internodes) and two genotypes (B47, *M. sativa* ssp. *sativa* and F56, *M. sativa* spp. *falcata*, Figure 3C). Six transcripts—namely, MSAD_243438 and MSAD_243443 (both *ATSPO11-1*), MSAD_214796 (*CYCA1;2*/*TAM*), MSAD_242787 and MSAD_308971 (both *SWI1*/*DYAD*), and MSAD_259662 (GIG1/*OSD1*)—showed the highest accumulation in flowers (Figure 3C, expression data are available in Table S3). Of particular importance was MSAD_259662, whose transcript levels were 10–300 times higher in the flowers of the two genotypes than in the rest of the tissues analyzed. Unfortunately, the RNA-seq data were limited to a whole flower at a single developmental stage, making it impossible to appreciate the expression level variations of these genes in different whorls. In these terms, a comprehensive expression atlas focused on flower tissues, and aimed at clarifying whether some of these transcripts are anther- or ovary-specific, should represent a primary goal in the study of the genes involved in unreduced gamete formation. A further aspect that should be evaluated is the variation in the expression of these candidate genes in both wild-type genotypes and genotypes capable of producing unreduced gametes. Finally, thanks to a well-established and efficient clustered regularly interspaced short palindromic repeats (CRISPR)/Cas9-based genome editing protocol recently published [91] that is able to precisely introduce tetra-allelic mutations into null mutants, it could be possible to evaluate the phenotypic effect related to the silencing of some of these genes.

## 4. Concluding Remarks and Future Perspectives

The natural capability of producing unreduced gametes in the genus *Medicago* is undoubtedly a fundamental resource for breeding new and superior varieties of cultivated alfalfa. Sexual polyploidization represents a direct way to exploit the introgression of useful traits from wild diploid relatives, and the combination of benefits resulting from both polyploidy and heterozygosity, as essential components for the expression of progressive heterosis.

Traditional breeding approaches lead to roughly consistent results. On the one hand, in the last twenty years, breeders have discovered and developed mutant lines of alfalfa able to produce a high proportion of unreduced gametes, especially tetraploid plants with diplosporic phenotypes; on the other hand, the case study reported here would suggest that apomeiosis maintained in sexually tetraploidized plants is developmentally independent from parthenogenesis. These findings suggest that alfalfa meiotic mutants show a null or a very low capacity for parthenogenesis of their unreduced egg cells. In this way, the powerful opportunity to assemble functional apomixis in alfalfa breeding programs in order to clone superior genotypes through seeds appears more challenging.

In this context, the genomic resources developed in recent years in alfalfa could represent a concrete alternative for overcoming this apparent impasse. Since the meiotic process is one of the most conserved biological phenomena in eukaryotes, it is highly likely that genes controlling meiosis and related mutations are homologous between different organisms. Therefore, a reverse genetics approach appears to be the most effective means of exploiting the genomic and transcriptomic resources available in alfalfa. To this aim, with an in silico approach, orthologous genes of nine *Arabidopsis thaliana* genic loci involved in unreduced gamete formation were identified in *Medicago* spp. We were able to demonstrate that some of them also showed high expression levels in flower tissues. Although more focused studies are needed in order to better appreciate the expression level variation of these genes in different whorls and in wild-type individuals and unreduced gamete producers, the availability of well-established and efficient genome editing protocols based on CRISPR /Cas9 represents a stepping stone, and is an unmissable opportunity to evaluate the phenotypic effects related to the silencing of some of these genes.

Next-generation resources including alfalfa genomes, flower-specific transcriptomes, and targeted gene editing platforms embody the key to extending the possibilities for deeper exploration of the genetic factors and molecular functions that lead to both FDR- and SDR-type megaspores, and a complete characterization of the whole biological process responsible for the production of unreduced gametes. In fact, the combination of well-established meiotic mutant lines with full knowledge of the master genes strictly involved in 2*n*-gamete production represents a strategy with great potential to be exploited for obtaining genetically stable varieties with improved agronomic traits.

The coexistence of elements of apomixis and sexuality in alfalfa plants suggests that both apomeiosis and parthenogenesis can be reversibly superimposed upon sexuality. Epigenetic control of apomixis is emerging in model species, where it is investigated with increasing support from studies on sexual plants wherein mutations of genes involved in epigenetic pathways resulted in phenotypes that mimic apomictic features ([58,108,109,110], and references therein). Despite the growing experimental evidence for epigenetic variations as important regulators of plant reproductive systems, epigenomics still has unexplored potential, and merits further focused studies to further the understanding of whether, and how, chromatin remodeling that does not entail any change to DNA sequences may affect the expression of apomixis and/or its components. Therefore, we are confident that in the near future apomixis could be introgressed and assembled into cultivated alfalfa by switching off the fundamental genes for sexuality to an apomixis-like pathway by transferring or editing the candidate gene(s).

## Figures and Tables

**Table 1:** Information on BC_1_ plants, including nuclear DNA content, ploidy level, and genetic similarities with 2*n* gamete producers, along with reproductive behavior (these data have been retrieved, computed, or adapted from [15,56]).

BC_1_ Plant Code	Nuclear DNA (pg)	Ploidy Level	Genetic Similarity		Observed Ovules	Diplospory (%)	Nucleolus Diameter (μm)		Self-Fertility	Mean Cross-Fertility	
			With TNE	With Pollinator			Meiotic	Diplosporic		With 2*x*	With 4*x*
A2	1.471	2*x*	0.992	0.137	98	15.31	1.53 ± 0.03	2.58 ± 0.13	0.009	0.190	0.036
B1	2.972	4*x*	0.608	0.451	132	19.70	1.56 ± 0.08	2.71 ± 0.11	0	0.008	0.935
B2	2.982	4*x*	0.549	0.510	203	21.67	1.45 ± 0.07	2.26 ± 0.12	0	0.006	0.848
B3	2.855	4*x*	0.431	0.627	164	0	−	−	0.022	0.019	1.466
B4	2.689	4*x*	0.686	0.373	110	0	−	−	0.018	0.014	1.523
B5	2.724	4*x*	0.725	0.333	108	13.89	1.31 ± 0.08	2.37 ± 0.15	0	0.041	0.530
B6	3.110	4*x*	0.588	0.471	94	2.13	−	−	0	0.019	0.796
B8	3.195	4*x*	0.667	0.392	83	40.96	1.38 ± 0.09	2.26 ± 0.10	0	0.018	0.645
C3	2.780	4*x*	0.529	0.529	151	15.23	1.81 ± 0.07	2.62 ± 0.19	0.016	0	0.964
C6	2.791	4*x*	0.471	0.588	119	5.04	1.58 ± 0.05	2.35 ± 0.10	0	0	0.511
C7	3.132	4*x*	0.511	0.549	112	6.25	1.44 ± 0.06	2.02 ± 0.05	0	0.010	0.677
C9	3.165	4*x*	0.509	0.549	96	3.13	−	−	0	0.024	1.233
D1	3.088	4*x*	0.618	0.400	208	19.23	1.55 ± 0.05	2.44 ± 0.11	0	0.019	1.446
D2	2.869	4*x*	0.618	0.364	98	0	−	−	0.089	0	2.071
D3	1.534	2*x*	0.909	0.145	95	57.89	1.39 ± 0.10	2.38 ± 0.09	0	0	0.538
E1	3.077	4*x*	0.709	0.309	137	17.52	1.53 ± 0.04	2.57 ± 0.13	0	0	1.479
F1	2.986	4*x*	0.618	0.364	123	2.44	1.60 ± 0.09	1.93 ± 0.07	0.021	0.011	0.401
F2	1.443	2*x*	0.891	0.164	54	7.41	1.52 ± 0.06	2.29 ± 0.14	0	0.720	0.022
DTA plants	2.961	4*x*	0.589	0.454	129	17.72	1.51 ± 0.07	2.40 ± 0.12	0.007	0.013	1.035

**Table 2:** Identification of candidate genes involved in unreduced gamete production in *M. sativa*. Nine proteins belonging to *A. thaliana* were selected for their direct involvement in unreduced male and/or female gamete production (column defect type) through first division restitution (FDR) or second division restitution (SDR) events. Forty-seven putative orthologs were then retrieved from the alfalfa genome [91] through a BLASTp alignment (chromosome locations of the related genes, Bit scores and E-values are reported). Finally, the corresponding transcripts were then searched through a BLASTp approach (Bit scores and E-values are reported in the last two columns), aligning the 47 proteins against the *M. sativa* atlas (available from [90]).

TAIR ID	Gene Name—TAIR	Type	Defect Type	Genomic Data				Transcriptomic Data		
				Gene Locus ID	Location	Bit	E-Value	Transcript	Bit	E-Value
AT5G51330	DYAD, SWI1, SWITCH1	FDR	♀	MS.gene066919	chr8.4	389	5 × 10^−124^	MSAD_308971, MSAD_242787	1481	0.0
				MS.gene59890	chr8.3	387	3 × 10^−123^		1279	0.0
				MS.gene016283	chr8.2	387	3 × 10^−123^		1279	0.0
				MS.gene067251	chr8.1	370	1 × 10^−116^		1438	0.0
AT1G34355	ATPS1, PARALLEL SPINDLE 1, PS1	FDR	♂	MS.gene006027	chr1.3	220	1 × 10^−57^	MSAD_236607, MSAD_225312	2228	0.0
				MS.gene041728	chr1.1	214	1 × 10^−55^		2414	0.0
				MS.gene33974	chr1.4	214	2 × 10^−55^		2410	0.0
				MS.gene60815	chr1.2	209	9 × 10^−54^		2357	0.0
AT1G06660	JASON	FDR	♂	MS.gene019180	chr3.3	254	1 × 10^−77^	MSAD_324822, MSAD_301063	915	0.0
				MS.gene000509	chr3.2	249	9 × 10^−76^		917	0.0
				MS.gene69498	chr3.1	246	1 × 10^−74^		915	0.0
				MS.gene22197	chr3.4	241	8 × 10^−73^		837	0.0
				MS.gene038276	chr5.4	244	2 × 10^−73^	MSAD_213698, MSAD_330161	974	0.0
				MS.gene47715	chr5.3	243	5 × 10^−73^		973	0.0
				MS.gene48029	chr5.2	242	1 × 10^−72^		975	0.0
				MS.gene017059	chr5.1	241	4 × 10^−72^		964	0.0
AT3G13170	ATSPO11-1, SPO11-1	FDR	♂/♀	MS.gene63906	chr5.1	520	0.0	MSAD_256423, MSAD_243443, MSAD_256426, MSAD_243438	746	0.0
AT5G05490	ATREC8, DETERMINATE INFERTILE 1, DIF1, REC8, SYN1, SYNAPTIC 1	FDR	♂/♀	MS.gene29556	chr5.2	364	2 × 10^−116^	MSAD_301520	1079	0.0
				MS.gene32484	chr5.1	363	7 × 10^−116^		1079	0.0
				MS.gene80251	chr5.3	338	4 × 10^−106^		1196	0.0
				MS.gene80593	chr5.4	330	3 × 10^−103^		1165	0.0
				MS.gene017435	chr5.1	326	1 × 10^−101^		1042	0.0
				MS.gene94220	chr5.4	335	4 × 10^−105^	MSAD_276303, MSAD_221382	1156	0.0
				MS.gene071227	chr5.1	334	9 × 10^−105^		1158	0.0
				MS.gene29681	chr5.3	333	2 × 10^−104^		1161	0.0
AT3G48750	CDKA;1, CELL DIVISION CONTROL 2	SDR	♂	MS.gene35086	chr4.4	520	0.0	MSAD_325109, MSAD_253844	610	0.0
				MS.gene28739	chr4.3	520	0.0		610	0.0
				MS.gene36424	chr4.1	520	0.0		610	0.0
				MS.gene028366	chr4.2	518	0.0		604	0.0
				MS.gene67884	chr2.4	520	0.0	MSAD_299311, MSAD_218596	610	0.0
				MS.gene55900	chr2.3	520	0.0		610	0.0
				MS.gene00063	chr2.1	520	0.0		610	0.0
AT1G77390	CYCA1;2, TAM, TARDY ASYNCHRONOUS MEIOSIS	SDR	♂/♀	MS.gene46260	chr3.1	421	5 × 10^−142^	MSAD_316334	987	0.0
				MS.gene013782	chr3.4	421	5 × 10^−142^		994	0.0
				MS.gene02568	chr3.2	420	2 × 10^−141^		1026	0.0
				MS.gene03197	chr3.3	400	4 × 10^−134^		986	0.0
				MS.gene02569	chr3.2	386	3 × 10^−128^	MSAD_316333, MSAD_214796	975	0.0
AT3G57860	GIG1, OMISSION OF SECOND DIVISION, OSD1	SDR	♂/♀	MS.gene017947	chr7.1	119	3 × 10^−31^	MSAD_259667, MSAD_259662	476	2 × 10^−171^
				MS.gene072366	chr7.4	115	5 × 10^−30^		478	1 × 10^−171^
				MS.gene50450	chr7.2	115	5 × 10^−30^		478	1 × 10^−171^
AT3G43210	ARABIDOPSIS NPK1-ACTIVATING KINESIN 2, ATNACK2, NACK2, TES, TETRASPORE, STUD	SDR	♂	MS.gene26619	chr8.4	1160	0.0	MSAD_280910, MSAD_270495	1908	0.0
				MS.gene26706	chr8.4	1158	0.0		1909	0.0
				MS.gene030239	chr8.2	1157	0.0		1966	0.0
				MS.gene012173	chr8.4	825	0.0		1483	0.0
				MS.gene042382	chr6.2	1054	0.0	MSAD_264334, MSAD_234901	1995	0.0
				MS.gene81904	chr6.4	1052	0.0		2004	0.0
				MS.gene05369	chr6.3	1050	0.0		1990	0.0

**Figure A1:**
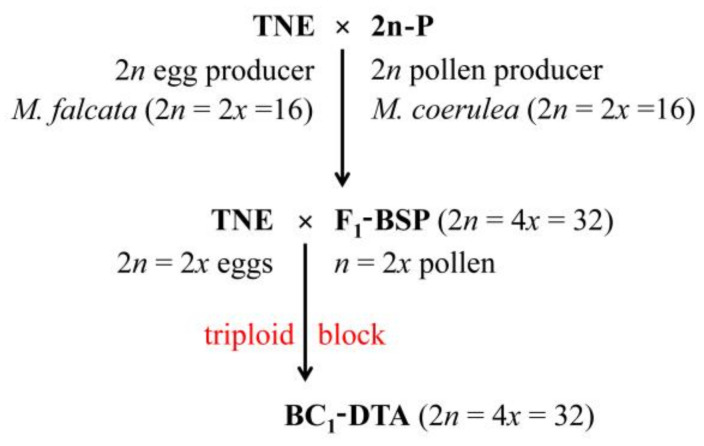
Strategy used for the introgression at the tetraploid level of the unreduced egg trait (TNE: diploid two—*n*—egg mutant; 2*n*–P: diploid 2*n*–pollen mutant; F_1_–BSP: tetraploidized hybrid progeny plants deriving from bilateral sexual polyploidization schemes; BC_1_–DTA: first generation backcross progeny including diplosporic tetraploidized alfalfa plants).

**Figure A2:**
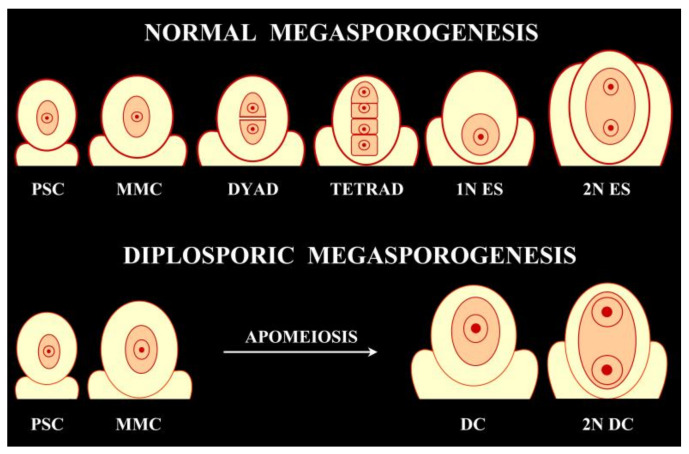
Developmental pathway of the primary sporogenic cell (PSC) and megaspore mother cell (MMC) during normal megasporogenesis (wild-type plants) and apomeiosis in the diplosporic TNE mutant. The diplosporic cells (DC) and binucleate embryo sacs (2N DC) were cytologically recognizable from sexual counterparts (1N ES and 2N ES) on the basis of both nucleolus diameter and integument length (for further details on the features of the apomeiotic mutant, see [19]).

## Data Availability

The data presented in this study are available as Supplementary Materials.

## Supplementary Materials

The following are available online at https://www.mdpi.com/article/10.3390/plants10050999/s1: Table S1: Results of wide-crosses and auxin treatments on DTA plants. Table S2: Identification of candidate genes involved in unreduced gamete production in *M. sativa.* Table S3: Relative abundance levels of 28 transcripts putatively corresponding to as many as 47 proteins, whose sequence information is reported in Table 2 and Table S2 (BLASTp; http://blast.ncbi.nlm.nih.gov/Blast.cgi, access on: 15/03/2021), evaluated in 6 different tissues (roots, root nodules, leaves, flowers, elongating stem internodes, and post-elongation stem internodes) and two genotypes (B47, *M. sativa* ssp. *sativa* and F56, *M. sativa* ssp. *falcata*) (data were retrieved from [90]).

## Appendix A

Both bilateral and unilateral sexual polyploidization schemes were adopted for introgressing the diplosporic mutation at the tetraploid level, by crossing the TNE mutant plants of *M. falcata* as female (2*n* egg cell producer) with several 2*n* pollen producer mutants of *M. coerulea* L. as male (for further details on plant materials see [56]). A total of 33 tetraploidized F_1_ plants obtained were backcrossed to TNE, employed as 2*n* egg cell donors, and then their progenies were screened for levels of ploidy and occurrence of diplospory (Figure A1). The BC_1_ plants were then selfed and crossed as females with diploid and tetraploid unrelated *Medicago* testers as males, using 30–50 flowers per mating combination. For each mating combination, seed set (SS) values were calculated and used to evaluate the single plant fertility, verify the egg cell viability, and provide an indication of their parthenogenetic capacity.

All BC_1_ plants were used to ascertain the nuclear DNA content via flow cytometry analysis. Nuclei isolation and purification was performed as in [55], along with the addition of the fluorochrome propidium iodide (PI) and RNase. Unstained chicken red blood cell (CRBC) nuclei were added as an internal standard. Nuclei suspensions were processed using a flow cytometer equipped with an argon-ion laser. The fluorescence emitted from PI-stained nuclei was collected through a 620/30 nm band-pass filter, and fluorescence pulses were acquired according to their height, width, and area. For each sample, the plant nuclear 2C DNA content, measured in picograms, was determined by taking the ratio of the G0/G1 peak mean and the red blood cell peak mean, and multiplying by the known CRBC nuclear DNA content (2.33 pg).

Stain-clearing techniques were used to quantify diplosporic tendencies. Inflorescences of BC_1_ plants, spanning the stages from archesporium formation to embryo sac maturation, were collected and fixed in FAA. Cytoembryological investigations of megasporogenesis and gametogenesis were carried out using the stain-clearing technique described by [111], with some modifications. Ovaries were dissected under a stereomicroscope, submitted to 2 h staining phase, mounted on slides with a drop of methyl salicylate, and examined under a light microscope. Overall, 54–208 ovaries from each selected plant were investigated. The degree of apomeiosis was calculated as the frequency of ovules with clear evidence of diplosporic cells or embryo sacs, at stages from the megaspore mother cell (MMC) to the four-nucleated embryo sac (Figure A2). Moreover, wide crosses were performed. To this aim, DTA plants were first emasculated, and then their inflorescences were cleaned from already-pollinated flowers and immature flowers, immersed in 50% alcohol solution, and then pollinated with pollen taken from unrelated diploid plants.

At the same time, some DTA plants were treated with three different auxin solutions, starting from the pre-meiotic stage. In particular, inflorescences of these plants were sprayed with 10 mg/L NAA (naphthalene acetic acid), 8 mg/L 2,4-D (2,4-dichlorophenoxyacetic acid), and 6 mg/L 2,4,5-T (2,4,5-trichlorophenoxyacetic acid). Treatments were repeated every two days until flowering.

Molecular DNA fingerprinting with RAPD and AFLP markers was used to discriminate plants of hybrid origin within the BC_1_ progeny and to assess their genetic similarity with respect to the parental mutants. PCR conditions reported by Barcaccia [112] and Barcaccia et al. [53] were used for RAPD fingerprinting with five 10-mer primers (OP-B9, OP-P3, OP-P10, OP-Q10, and OP-R10). AFLP fingerprinting was performed according to Barcaccia et al. [113], using two different *Eco*-RI/*Mse*-I primer combinations with three selective nucleotides (CCA/AGA and CAC/AGT). Genetic similarity estimates between each pairwise comparison of diploid parental plants and BC_1_ progeny plants were assessed by calculating the simple matching coefficients, after excluding markers that were shared between parents and were non-segregating in the progenies.

## References

[1] Mendiburu A.O., Peloquin S.J. (1976). Sexual polyploidization and depolyploidization: Some terminology and definitions. Theor. Appl. Genet..

[2] De Wet J.M. (1980). Origins of polyploids. Basic Life Sci..

[3] Darlington C. (1973). Chromosome Botany and the Origins of Cultivated Plants.

[4] Harlan J.R., de Wet J.M.J. (1975). On Ö. Winge and a Prayer: The origins of polyploidy. Bot. Rev..

[5] Lesins K.A., Lesins I. (1979). Genus Medicago a Taxogenetic Study.

[6] Calderini O., Pupilli F., Cluster P.D., Mariani A., Arcioni S. (1996). Cytological studies of the nucleolus organizing regions in the *Medicago* complex: *Sativa-coerulea-falcata*. Genome.

[7] Stanford E.H., Clement W.M., Bingham E.T. (2015). Cytology and Evolution of the *Medicago sativa-falcata* Complex.

[8] Bingham E.T. (1979). Maximizing heterozygosity in autopolyploids. Basic Life Sci..

[9] Hermsen J.G.T. (1984). The potential of meiotic polyploidization in breading allogamous crops. Iowa State J. Res..

[10] Veronesi F., Mariani A., Bingham E.T. (1986). Unreduced gametes in diploid *Medicago* and their importance in alfalfa breeding. Theor. Appl. Genet..

[11] McCoy T.J., Bingham E.T. (1988). Cytology and Cytogenetics of Alfalfa.

[12] McCoy T.J., Tavoletti S., Mariani A. (1992). Genome manipulation and molecular genetic analysis of alfalfa (*Medicago sativa*). Gametes with Somatic Chromosome Number in the Evolution and Breeding of Polyploid Polysomic Species: Achievements and Perspectives.

[13] Barcaccia G., Tavoletti S., Mariani A., Veronesi F. (2003). Occurrence, inheritance and use of reproductive mutants in alfalfa improvement. Euphytica.

[14] McCoy T.J., Rowe D.E. (1986). Single cross alfalfa (*Medicago sativa* L.) hybrids produced via 2n gametes and somatic chromosome doubling: Experimental and theoretical comparisons. Theor. Appl. Genet..

[15] Barcaccia G., Albertini E., Luchin M., Veronesi F., Falcinelli M., Rosellini D. (1999). Progress in assembling a functional system of apomictic seed production in alfalfa. Herbage Seed as a Key Factor for Improving Production and Environmental Quality.

[16] Vorsa N., Bingham E.T. (1979). Cytology of 2n pollen formation in diploid alfalfa, *Medicago sativa*. Can. J. Genet. Cytol..

[17] Tavoletti S. (1994). Cytological mechanisms of 2n egg formation in a diploid genotype of *Medicago sativa* subsp. *falcata*. Euphytica.

[18] McCoy T.J. (1982). The inheritance of 2n pollen formation in diploid alfalfa *Medicago sativa*. Can. J. Genet. Cytol..

[19] Barcaccia G., Tavoletti S., Falcinelli M., Veronesi F. (1997). Environmental influences on the frequency and viability of meiotic and apomeiotic cells of a diploid mutant of alfalfa. Crop Sci..

[20] Peloquin S.J., Mulcahy D.L., Mulcahy G.B., Ottaviano E. (1985). GChromosome Engineering with Meiotic Mutants. Biotechnology and Ecology of Pollen.

[21] Kaul M.L.H., Murthy T.G.K. (1985). Mutant genes affecting higher plant meiosis. Theor. Appl. Genet..

[22] Clement W.M., Stanford E.H. (1961). A mechanism for the production of tetraploid and pentaploid progeny from diploid × tetraploid crosses of alfalfa. Crop Sci..

[23] Tavoletti S., Mariani A., Veronesi F. (1991). Cytological analysis of macro- and microsporogenesis of a diploid alfalfa clone producing male and female 2n gametes. Crop Sci..

[24] McCoy T.J., Smith L.Y. (1983). Genetics, cytology, and crossing behavior of an alfalfa (*Medicago sativa*) mutant resulting in failure of the postmeiotic cytokinesis. Can. J. Genet. Cytol..

[25] Pfeiffer T.W., Bingham E.T. (1983). Abnormal meiosis in alfalfa, *Medicago sativa*: Cytology of 2 N egg and 4 N pollen formation. Can. J. Genet. Cytol..

[26] Barcaccia G., Mazzuccato A., Falcinelli M., Veronesi F. (1996). Callose localization during meiotic and apomeiotic megasporo- genesis in alfalfa (*Medicago* ssp.). Caryologia.

[27] Tavoletti S., Bingham E.T., Yandell B.S., Veronesit F., Osborn T.C. (1996). Half tetrad analysis in alfalfa using multiple restriction fragment length polymorphism markers. Proc. Natl. Acad. Sci. USA.

[28] Johnston S.A., Den Nijs T.P.M., Peloquin S.J., Hanneman R.E. (1980). The significance of genic balance to endosperm development in interspecific crosses. Theor. Appl. Genet..

[29] Calderini O., Mariani A. (1994). Identification of meiotic mutants producing 2n pollen in the *Medicago sativa* complex. J. Genet. Breed..

[30] Comai L. (2005). The advantages and disadvantages of being polyploid. Nat. Rev. Genet..

[31] Mayrose I., Zhan S.H., Rothfels C.J., Magnuson-Ford K., Barker M.S., Rieseberg L.H., Otto S.P. (2011). Recently formed polyploid plants diversify at lower rates. Science.

[32] Mason A.S., Pires J.C. (2015). Unreduced gametes: Meiotic mishap or evolutionary mechanism?. Trends Genet..

[33] Soltis D.E., Segovia-Salcedo M.C., Jordon-Thaden I., Majure L., Miles N.M., Mavrodiev E.V., Mei W., Cortez M.B., Soltis P.S., Gitzendanner M.A. (2014). Are polyploids really evolutionary dead-ends (again)? A critical reappraisal of Mayrose. New Phytol..

[34] Madlung A. (2013). Polyploidy and its effect on evolutionary success: Old questions revisited with new tools. Heredity.

[35] Cuypers T.D., Hogeweg P. (2014). A Synergism between Adaptive Effects and Evolvability Drives Whole Genome Duplication to Fixation. PLoS Comput. Biol..

[36] Mable B.K., Otto S.P. (2001). Masking and purging mutations following EMS treatment in haploid, diploid and tetraploid yeast (*Saccharomyces cerevisiae*). Genet. Res..

[37] Stadler L.J. (1929). Chromosome number and the mutation rate in *Avena* and *Triticum*. Proc. Natl. Acad. Sci. USA.

[38] Singh R.J. (2003). Plant Cytogenetics.

[39] Stupar R.M., Bhaskar P.B., Yandell B.S., Rensink W.A., Hart A.L., Ouyang S., Veilleux R.E., Busse J.S., Erhardt R.J., Buell C.R. (2007). Phenotypic and transcriptomic changes associated with potato autopolyploidization. Genetics.

[40] Allario T., Brumos J., Colmenero-Flores J.M., Tadeo F., Froelicher Y., Talon M., Navarro L., Ollitrault P., Morillon R. (2011). Large changes in anatomy and physiology between diploid Rangpur lime (*Citrus limonia*) and its autotetraploid are not associated with large changes in leaf gene expression. J. Exp. Bot..

[41] Aversano R., Scarano M.T., Aronne G., Caruso I., D’Amelia V., De Micco V., Fasano C., Termolino P., Carputo D. (2015). Genotype-specific changes associated to early synthesis of autotetraploids in wild potato species. Euphytica.

[42] Innes L.A., Denton M.D., Dundas I.S., Peck D.M., Humphries A.W. (2020). The effect of ploidy number on vigor, productivity, and potential adaptation to climate change in annual *Medicago* species. Crop Sci..

[43] Anssour S., Krügel T., Sharbel T.F., Saluz H.P., Bonaventure G., Baldwin I.T. (2009). Phenotypic, genetic and genomic consequences of natural and synthetic polyploidization of *Nicotiana attenuata* and *Nicotiana obtusifolia*. Ann. Bot..

[44] Riddle N.C., Jiang H., An L., Doerge R.W., Birchler J.A. (2010). Gene expression analysis at the intersection of ploidy and hybridity in maize. Theor. Appl. Genet..

[45] Ramanna M.S., Jacobsen E. (2003). Relevance of sexual polyploidization for crop improvement—A review. Euphytica.

[46] Obajimi A.O., Bingham E.T. (1973). Inbreeding cultivated alfalfa in one tetraploid-haploid-tetraploid cycle: Effects on morphology, fertility, and cytology. Crop Sci..

[47] Hahn M.A., Lanz T., Fasel D., Müller-Schärer H. (2013). Increased seed survival and seedling emergence in a polyploid plant invader. Am. J. Bot..

[48] Rosellini D., Ferradini N., Allegrucci S., Capomaccio S., Zago E.D., Leonetti P., Balech B., Aversano R., Carputo D., Reale L. (2016). Sexual polyploidization in *Medicago sativa* L.: Impact on the phenotype, gene transcription, and genome methylation. G3 Genes Genomes Genet..

[49] Bond W.J., Honig M., Maze K.E. (1999). Seed size and seedling emergence: An allometric relationship and some ecological implications. Oecologia.

[50] Wang J., Tian L., Lee H.-S., Wei N.E., Jiang H., Watson B., Madlung A., Osborn T.C., Doerge R.W., Comai L. (2006). Genomewide nonadditive gene regulation in *Arabidopsis* allotetraploids. Genetics.

[51] Bingham E.T., Lewis W.H. (1980). Maximizing heterozygosity in autotetraptoids. Polyploidy: Biological Relevance.

[52] Bingham E.T. (1971). Isolation of haploids of tetraploid alfalfa. Crop Sci..

[53] Barcaccia G., Tavoletti S., Falcinelli M., Veronesi F. (1997). Verification of the parthenogenetic capability of unreduced eggs in an alfalfa mutant by a progeny test based on morphological and molecular markers. Plant Breed..

[54] Asker S., Jerling L. (1992). Apomixis in Plants.

[55] Lucretti S., Doležel J., Galbraith D.W., Bohnert H., Bourque D.P. (1995). Cell cycle synchronization, chromosome isolation, and flow-sorting in plants. Methods in Plant Cell Biology.

[56] Albertini E. (2000). Investigation and manipolation of reproductive systems in *Medicago* spp. and *Poa pratensis* L.. Ph.D. Thesis.

[57] Barcaccia G., Albertini E., Falcinelli M. (1999). AFLP fingerprinting in *Pelargonium peltatum*: Its development and potential in cultivar identification. J. Hortic. Sci. Biotechnol..

[58] Barcaccia G., Palumbo F., Sgorbati S., Albertini E., Pupilli F. (2020). A reappraisal of the evolutionary and developmental pathway of apomixis and its genetic control in angiosperms. Genes.

[59] Oleszczuk S., Grzechnik N., Mason A.S., Zimny J. (2019). Heritability of meiotic restitution and fertility restoration in haploid triticale. Plant Cell Rep..

[60] Veronesi F., Mariani A., Tavoletti S. (1988). Screening for 2N gamete producers in diploid species of genus *Medicago*. Genet. Agrar..

[61] Brownfield L., Köhler C. (2011). Unreduced gamete formation in plants: Mechanisms and prospects. J. Exp. Bot..

[62] Lesins K.A., Lesins I., Junk W. (1979). Genus *Medicago* (Leguminosae). A Taxogenetic Study.

[63] Diwan N., Bouton J.H., Kochert G., Cregan P.B. (2000). Mapping of simple sequence repeat (SSR) DNA markers in diploid and tetraploid alfalfa. Theor. Appl. Genet..

[64] Brouwer D.J., Osborn T.C. (1999). A molecular marker linkage map of tetraploid alfalfa (*Medicago sativa* L.). Theor. Appl. Genet..

[65] Kiss G.B., Csanádi G., Kálmán K., Kaló P., Ökrész L. (1993). Construction of a basic genetic map for alfalfa using RFLP, RAPD, isozyme and morphological markers. Mol. Gen. Genet..

[66] Echt C.S., Kidwell K.K., Knapp S.J., Osborn T.C., McCoy T.J. (1994). Linkage mapping in diploid alfalfa (*Medicago sativa*). Genome.

[67] Tavoletti S., Pesaresi P., Barcaccia G., Albertini E., Veronesi F. (2000). Mapping the jp (jumbo pollen) gene and QTLs involved in multinucleate microspore formation in diploid alfalfa. Theor. Appl. Genet..

[68] Barcaccia G., Albertini E., Rosellini D., Tavoletti S., Veronesi F. (2000). Inheritance and mapping of 2n-egg production in diploid alfalfa. Genome.

[69] Barcaccia G., Varotto S., Meneghetti S., Albertini E., Porceddu A., Parrini P., Lucchin M. (2001). Analysis of gene expression during flowering in apomeiotic mutants of *Medicago* spp.: Cloning of ESTs and candidate genes for 2n eggs. Sex. Plant Reprod..

[70] Zamariola L., Tiang C.L., De Storme N., Pawlowski W., Geelen D. (2014). Chromosome segregation in plant meiosis. Front. Plant Sci..

[71] Silkova O.G., Loginova D.B. (2016). Sister chromatid separation and monopolar spindle organization in the first meiosis as two mechanisms of unreduced gametes formation in wheat–rye hybrids. Plant Reprod..

[72] Wang J., Kang X., Zhu Q. (2010). Variation in pollen formation and its cytological mechanism in an allotriploid white poplar. Tree Genet. Genomes.

[73] Mariani A., Campanoni P., Gianì S., Breviario D. (2000). Meiotic mutants of Medicago sativa show altered levels of α- and β-tubulin. Genome.

[74] Barcaccia G., Varotto S., Albertini E., Parrini P., Lucchin M. MOB (Mps-one-binder), a multi gene family that may shed light on apomeiosis in alfalfa mutants. Proceedings of the XVII Int Congress on Sexual Plant Reproduction.

[75] Luca F.C., Winey M. (1998). Mob1, an essential yeast gene required for completion of mitosis and maintenance of ploidy. Mol. Biol. Cell.

[76] Luca F.C., Mody M., Kurischko C., Roof D.M., Giddings T.H., Winey M. (2001). *Saccharomyces cerevisiae* Mob1p Is Required for Cytokinesis and Mitotic Exit. Mol. Cell. Biol..

[77] Chow A., Hao Y., Yang X. (2010). Molecular characterization of human homologs of yeast MOB1. Int. J. Cancer.

[78] Citterio S., Albertini E., Varotto S., Feltrin E., Soattin M., Marconi G., Sgorbati S., Lucchin M., Barcaccia G. (2005). Alfalfa Mob1-like genes are expressed in reproductive organs during meiosis and gametogenesis. Plant Mol. Biol..

[79] Citterio S., Piatti S., Albertini E., Aina R., Varotto S., Barcaccia G. (2006). Alfalfa Mob1-like proteins are involved in cell proliferation and are localized in the cell division plane during cytokinesis. Exp. Cell Res..

[80] Yang S.S., Xu W.W., Tesfaye M., Lamb J.F.S., Jung H.G., Samac D.A., Vance C.P., Gronwald J.W. (2009). Single-Feature Polymorphism Discovery in the Transcriptome of Tetraploid Alfalfa. Plant Genome.

[81] Li X., Acharya A., Farmer A.D., Crow J.A., Bharti A.K., Kramer R.S., Wei Y., Han Y., Gou J., May G.D. (2012). Prevalence of single nucleotide polymorphism among 27 diverse alfalfa genotypes as assessed by transcriptome sequencing. BMC Genom..

[82] Li X., Han Y., Wei Y., Acharya A., Farmer A.D., Ho J., Monteros M.J., Brummer E.C. (2014). Development of an alfalfa SNP array and its use to evaluate patterns of population structure and linkage disequilibrium. PLoS ONE.

[83] Song L., Jiang L., Chen Y., Shu Y., Bai Y., Guo C. (2016). Deep-sequencing transcriptome analysis of field-grown *Medicago sativa* L. crown buds acclimated to freezing stress. Funct. Integr. Genomics.

[84] Zeng N., Yang Z., Zhang Z., Hu L., Chen L. (2019). Comparative transcriptome combined with proteome analyses revealed key factors involved in alfalfa (*Medicago sativa*) response to waterlogging stress. Int. J. Mol. Sci..

[85] Lei Y., Xu Y., Hettenhausen C., Lu C., Shen G., Zhang C., Li J., Song J., Lin H., Wu J. (2018). Comparative analysis of alfalfa (*Medicago sativa* L.) leaf transcriptomes reveals genotype-specific salt tolerance mechanisms. BMC Plant Biol..

[86] Liu W., Xiong C., Yan L., Zhang Z., Ma L., Wang Y., Liu Y., Liu Z. (2017). Transcriptome analyses reveal candidate genes potentially involved in al stress response in alfalfa. Front. Plant Sci..

[87] Vieira P., Mowery J., Eisenback J.D., Shao J., Nemchinov L.G. (2019). Cellular and transcriptional responses of resistant and susceptible cultivars of alfalfa to the root lesion nematode, *Pratylenchus penetrans*. Front. Plant Sci..

[88] Zhang S., Shi Y., Cheng N., Du H., Fan W., Wang C. (2015). De novo characterization of fall dormant and nondormant alfalfa (*Medicago sativa* L) leaf transcriptome and identification of candidate genes related to fall dormancy. PLoS ONE.

[89] Yang S.S., Tu Z.J., Cheung F., Xu W.W., Lamb J.A.F.S., Jung H.J.G., Vance C.P., Gronwald J.W. (2011). Using RNA-Seq for gene identification, polymorphism detection and transcript profiling in two alfalfa genotypes with divergent cell wall composition in stems. BMC Genom..

[90] O’Rourke J.A., Fu F., Bucciarelli B., Yang S.S., Samac D.A., Lamb J.A.F.S., Monteros M.J., Graham M.A., Gronwald J.W., Krom N. (2015). The *Medicago sativa* gene index 1.2: A web-accessible gene expression atlas for investigating expression differences between *Medicago sativa* subspecies. BMC Genom..

[91] Chen H., Zeng Y., Yang Y., Huang L., Tang B., Zhang H., Hao F., Liu W., Li Y., Liu Y. (2020). Allele-aware chromosome-level genome assembly and efficient transgene-free genome editing for the autotetraploid cultivated alfalfa. Nat. Commun..

[92] Pillet J., Yu H.W., Chambers A.H., Whitaker V.M., Folta K.M. (2015). Identification of candidate flavonoid pathway genes using transcriptome correlation network analysis in ripe strawberry (*Fragaria × ananassa*) fruits. J. Exp. Bot..

[93] Zhang S., Chen Y., He X., Du J., Zhang R., Ma Y., Hu X., Zhang Z., Chen Q., Wan X. (2020). Identification of MYB Transcription Factors Regulating Theanine Biosynthesis in Tea Plant Using Omics-Based Gene Coexpression Analysis. J. Agric. Food Chem..

[94] Barcaccia G., Palumbo F., Scariolo F., Vannozzi A., Borin M., Bona S. (2020). Potentials and challenges of genomics for breeding *Cannabis* Cultivars. Front. Plant Sci..

[95] Yang C., Hamamura Y., Sofroni K., Böwer F., Stolze S.C., Nakagami H., Schnittger A. (2019). SWITCH 1/DYAD is a WINGS APART-LIKE antagonist that maintains sister chromatid cohesion in meiosis. Nat. Commun..

[96] Agashe B., Prasad C.K., Siddiqi I. (2002). Identification and analysis of DYAD: A gene required for meiotic chromosome organisation and female meiotic progression in *Arabidopsis*. Development.

[97] Ravi M., Marimuthu M.P.A., Siddiqi I. (2008). Gamete formation without meiosis in Arabidopsis. Nature.

[98] Grelon M., Vezon D., Gendrot G., Pelletier G. (2001). AtSPO11-1 is necessary for efficient meiotic recombination in plants. EMBO J..

[99] Chelysheva L., Diallo S., Vezon D., Gendrot G., Vrielynck N., Belcram K., Rocques N., Márquez-Lema A., Bhatt A.M., Horlow C. (2005). AtREC8 and AtSCC3 are essential to the monopolar orientation of the kinetochores during meiosis. J. Cell Sci..

[100] D’Erfurth I., Jolivet S., Froger N., Catrice O., Novatchkova M., Simon M., Jenczewski E., Mercier R. (2008). Mutations in AtPS1 (*Arabidopsis thaliana* Parallel Spindle 1) lead to the production of diploid pollen grains. PLoS Genet..

[101] De Storme N., Geelen D. (2011). The *Arabidopsis* mutant jason produces unreduced first division restitution male gametes through a parallel/ fused spindle mechanism in meiosis II. Plant Physiol..

[102] Erilova A., Brownfield L., Exner V., Rosa M., Twell D., Scheid O.M., Hennig L., Köhler C. (2009). Imprinting of the Polycomb group gene MEDEA serves as a ploidy sensor in *Arabidopsis*. PLoS Genet..

[103] D’Erfurth I., Cromer L., Jolivet S., Girard C., Horlow C., Sun Y., To J.P.C., Berchowitz L.E., Copenhaver G.P., Mercier R. (2010). The CYCLIN-A CYCA1;2/TAM is required for the meiosis I to meiosis II transition and cooperates with OSD1 for the prophase to first meiotic division transition. PLoS Genet..

[104] Wang Y., Jha A.K., Chen R., Doonan J.H., Yang M. (2010). Polyploidy-associated genomic instability in *Arabidopsis thaliana*. Genesis.

[105] Dissmeyer N., Nowack M.K., Pusch S., Stals H., Inzé D., Grini P.E., Schnittger A. (2007). T-loop phosphorylation of *Arabidopsis* CDKA;1 is required for its function and can be partially substituted by an aspartate residue. Plant Cell.

[106] D’Erfurth I., Jolivet S., Froger N., Catrice O., Novatchkova M., Mercier R. (2009). Turning meiosis into mitosis. PLoS Biol..

[107] Yang C.Y., Spielman M., Coles J.P., Li Y., Ghelani S., Bourdon V., Brown R.C., Lemmon B.E., Scott R.J., Dickinson H.G. (2003). TETRASPORE encodes a kinesin required for male meiotic cytokinesis in *Arabidopsis*. Plant J..

[108] Pupilli F., Barcaccia G. (2012). Cloning plants by seeds: Inheritance models and candidate genes to increase fundamental knowledge for engineering apomixis in sexual crops. J. Biotechnol..

[109] Albertini E., Barcaccia G., Carman J.G., Pupilli F. (2019). Did apomixis evolve from sex or was it the other way around?. J. Exp. Bot..

[110] Podio M., Cáceres M.E., Samoluk S.S., Seijo J.G., Pessino S.C., Ortiz J.P.A., Pupilli F. (2014). A methylation status analysis of the apomixis-specific region in *Paspalum* spp. suggests an epigenetic control of parthenogenesis. J. Exp. Bot..

[111] Stelly D., Peloquin S.J., Palmer R.G., Crane C.F. (1984). Mayer’s hemalum-methyl salicylate: A stain-clearing technique for observations within whole ovules. Stain Technol..

[112] Barcaccia G. (1994). Development, comparability and potential applications of RAPD markers in the genus *Medicago*. J. Genet. Breed..

[113] Barcaccia G., Mazzucato A., Albertini E., Zethof J., Gerats A., Pezzotti M., Falcinelli M. (1998). Inheritance of parthenogenesis in *Poa pratensis* L.: Auxin test and AFLP linkage analyses support monogenic control. Theor. Appl. Genet..

